# Diagnosis of Early Pregnancy, with Reference to a Particular Sign

**Published:** 1907-06

**Authors:** 


### DIAGNOSIS OF EARLY PREGNANCY, WITH REFERENCE TO A PARTICULAR SIGN.

Ladinski is convinced that a diagnosis of early uterine pregnancy can be made or excluded in nearly every instance with almost absolute certainty. The probable signs of pregnancy in the early months are: (1) Changes in the shape and consistency of the body of the uterus; (2) increase in size of uterus; (3) changes in the cervix; (4) intermittent contractions of uterus. Of these signs the last two, namely, changes in the cervix and intermittent contractions of the uterus, can be looked upon any as corroborative signs. The author has found, however, that the change in the consistency of the uterus is an invariably constant and positive sign of early pregnancy; and, furthermore, that this change can be detected frequently as early as the fifth week, but always in the sixth week of pregnancy. As an indication of pregnancy it is in his opinion equally as reliable as any of the positive signs of advanced pregnancy, such as fœtal heart beat, passive and active movement of the fœtus, and outline of the fœtus. This change can be felt in the median line in the anterior wall of the body of the uterus just above the junction of the body and cervix; in other words, in the isthmus of the uterus, a circular area the size of the tip of the finger, which presents to the palpating finger the sensation of an elastic fluctuation. As pregnancy advances this area increases in size in a crescentic manner, until between the third and fourth month, when nearly the entire anterior body, with the exception of the upper crescent of the fundus, partakes of this change, and gives the cystic fluctuating feel to the examining finger. The change appears in the anterior wall of the uterus when the uterus is in the normal position or slightly anteverted, but in extremely retroverted or retroflexed uteri the elastic area appears in the posterior wall, but instead of being perceptible in the fifth or sixth week of pregnancy, is usually felt in the sixth or seventh week. As to the manner of obtaining this sign, it is absolutely essential that bimanual palpation be employed: While the external hand fixes the uterus by counterpressure, and the anterior wall of the uterus from the cervix to the fundus is palpated with the internal finger or fingers, the elastic area in the body of the uterus, immediately above the cervix, will be readily made out; the size of the area will correspond with the duration of pregnancy. The recognition of this elastic area does not require any special skill or dexterity; the ordinary technique and tactile sense requisite for a bimanual examination, combined with a correct interpretation of the change noted in the wall of the uterus, will enable the general practitioner to make a diagnosis with the same ease as the specialist. Again, the absence of this sign is an absolute indication for the exclusion of uterine pregnancy. In the absence of this change in the uterine wall, pregnancy of more than five or six weeks can be positively eliminated, even in the presence of other presumptive or probable signs. The author believes that the peculiar elasticity found in early pregnancy is in all probability due to the extreme vascularity of that particular portion of the uterine wall, and to no other cause; but later, however, the flucturating feel of the body of the uterus is no doubt due also to the presence of the gravid sac.—Medical Record, April 13th, 1907.

